# Source country perceptions, experiences, and recommendations regarding health workforce migration: a case study from the Philippines

**DOI:** 10.1186/1478-4491-12-62

**Published:** 2014-10-31

**Authors:** Kanchan Marcus, Gabriella Quimson, Stephanie D Short

**Affiliations:** Health Systems, Global Populations Faculty Research Group, Faculty of Health Sciences, The University of Sydney, Science Road, Sydney, NSW 2006 Australia; Integrity and Anticorruption Programme, Institute for Ethics Governance and Law, Griffith University, Nathan, Queensland 4111 Australia; Principal Adviser Integrity Philippine Department of Justice Witness Protection Programme, Department of Justice, Padre Faura Street, Ermita, Metro Manila Philippines; Discipline of Behavioural & Social Sciences in Health, Faculty of Health Sciences, The University of Sydney, Science Road, Sydney, NSW 2006 Australia

**Keywords:** Health workforce migration, Nurse migration, Philippines

## Abstract

**Background:**

The Philippines continues to overproduce nurses for export. Little first-hand evidence exists from leading organisations in the Philippines concerning their experiences and perceptions in relation to Filipino nurse migration. What are their views about health workforce migration? This paper addresses this research gap by providing a source country perspective on Filipino nurse migration to Australia.

**Methods:**

Focus-group interviews were conducted with key informants from nine Filipino organisations in the Philippines by an Australian-Filipino research team. The organisations were purposively selected and contacted in person, by phone, and/or email. Qualitative thematic analysis was performed using a coding framework.

**Results:**

Health workforce migration is perceived to have both positive and negative consequences. On the one hand, emigration offers a welcome opportunity for individual Filipino nurses to migrate abroad in order to achieve economic, professional, lifestyle, and social benefits. On the other, as senior and experienced nurses are attracted overseas, this results in the maldistribution of health workers particularly affecting rural health outcomes for people in developing countries. Problems such as ‘volunteerism’ also emerged in our study.

**Conclusions:**

In the context of the WHO (2010) Code of Practice on the International Recruitment of Health Personnel it is to be hoped that, in the future, government recruiters, managers, and nursing leaders can utilise these insights in designing recruitment, orientation, and support programmes for migrant nurses that are more sensitive to the experience of the Philippines’ education and health sectors and their needs.

## Background

There is a global shortage of approximately 2.3 million physicians, nurses, and midwives with 36 countries in sub-Saharan Africa experiencing critical shortages [1]. The global mobility of nurses is well documented [2–6] and the pattern of health workforce migration has become increasingly dynamic involving several countries. Reliance on Filipino nurses is an international phenomenon, and the demand for nursing services is predicted to exceed supply by almost 30% in 2020 in the USA [7]. Draining highly skilled professionals, typically from a developing country, negatively impacts health outcomes in developing countries through the maldistribution of professionals and an inadequate skill mix of health professionals. Local shortages of health workers in developing countries are worsened when allied health and medical professionals retrain as nurses in order to work abroad. Lack of experienced health professionals in the home country also jeopardises patient safety and health, thereby reducing the likelihood for developing nations to attain the Millennium Development Goals. Aging populations and workforce in developed countries pull nurses out of developing countries such as the Philippines, which purposely overproduces nurses for export. As a consequence, the Philippines is the major supplier of nurses worldwide.

This study provides an immigrant source country perspective, namely the Philippines, on health workforce migration. This research provides new insights into the current health worker situation in the Philippines in light of the global financial crisis. Filipino organisations provide first-hand perspectives regarding experiences and consequences in relation to nurse migration in particular. This study complements surveys, interviews, and ethical model analysis conducted in Queensland, Australia, as part of our larger project on ethical and sustainable health professional recruitment [8–10].

### The Philippines political economy

Historically, population growth in the Philippines has exceeded economic growth which has resulted in a shortage of job vacancies, high unemployment rates, and financial wage problems. Governance challenges have also impeded growth of the economy. During the Marcos era (1965–1986), the Philippine Overseas Employment Program was initiated as a temporary economic measure in order to address the high unemployment rate through encouraging Filipino employment within the global labour market. However, what started as a temporary solution became a deliberate national policy strategy [11]. During 2002 to 2004, civil service salaries did not increase, primary expenditure declined by 2% of the GDP, and spending on health also declined as a share of the GDP [12]. Remittances allowed for growth of the Philippines economy and as of December 2004, nearly 10% of the population were working and/or residing overseas. Despite annual remittances which contributed 10% to the Philippines GDP, poverty rates increased from 24.9% to 26.5% from 2003 to 2009 [13].

The global financial crisis of 2007 and 2008 and the subsequent reduction in the number of nursing graduates absorbed internationally resulted in an increase in local nursing graduates without job opportunities in the Philippines. The impact of nurse volunteerism expanded throughout hospitals, whereby graduates were employed without pay. Doctors also retrained as nurses in order to improve their prospects of migration. Disparities between the production of health professionals and the capacity for employment grew following the global financial crisis. In 2011, the Philippines almost reached a population of 100 million inhabitants, with a per-capita Gross National Income of US$ 2,210 and in the same year the Philippines’ Department of Health committed to resolving the oversupply issue by employing more than 11,500 nurses and 1,000 midwives on a program to encourage skills training and experience in order to increase the likelihood of paid employment [14]. This was also intended to prevent nurses moving into different professions, which results in skills wastage.

According to the International Labor Office [15], in 2010, an estimated 7.2 million Filipinos were working abroad with $21.3 US billion in remittances [16], draining the home country of skilled workers. Vast literature confirms the economic-financial drive to migrate abroad [17], where ‘pull’ factors, higher income, better lifestyle, travel opportunities, and career development pull nurses out of developed countries and push factors of low wages, increased workload, and poor benefits encourage Filipino nurses to exit their home country.

The socio-cultural impacts of health workforce migration upon developing countries like the Philippines are now coming to light as the gender distribution of health workers has shifted. Women have become breadwinners, despite the increasing number of men joining the nursing occupation. The role of women has evolved to support and provide for the entire family, which usually includes first and second cousins. Remittances affect socioeconomic status and the health of immigrants in the destination country and their families. There are macro and micro socio-cul-tural costs of migration, where migrant health workers are less likely to seek health services, express fear in losing their job, destabilising the family financially, and be positioned in regional or rural locations. According to Forrester [18], overseas-trained nurses are more likely to be placed in rural and remote settings in Australia, where there is little capacity for professional support and mentoring. The significance of socioeconomic-cultural barriers and health inequalities faced by migrants is attracting increased academic attention [19].

### Philippines-Australia context

Australia relies on foreign-born health workers, including medical practitioners and nurses [20]. An aging population and workforce will inevitably increase demand for services, especially in remote areas, and coupled with the current and predicted workforce shortages, they amplify the need for workforce sustainability. Australia and the Philippines do not yet share reciprocal agreements of any sort, and, therefore, there are limited opportunities for migration. Most nurses from the Philippines arrive in Australia as skilled migrants or under the family migration program. In 2009–2010, the Philippines was the fourth largest cohort of Family Stream migrants to Australia, behind China, the United Kingdom, and India [21]. Migrant Filipino workers contribute significantly to the Australian workforce with their labour force participation rate at 74%, which was well above the national average of 65% in 2011 [21]. From 2001 to 2006, over 1,000 Filipino degree-qualified nurses migrated to Australia, making the Philippines the second main source country after the United Kingdom [9]. Although Australian health workforce recruitment is preferred from other developed countries like Ireland and the United Kingdom in order to reduce the impact of brain circulation, these developed countries then recruit their nursing workforce from countries like the Philippines. The global cycle of international health workers is unavoidable, but source countries lose out on achieving millennium development goals, and are at increased risk of poor health outcomes.

This study was designed and conducted in light of the WHO (2010) Code of Practice on the International Recruitment of Health Personnel that was adopted by member states (Australia is a signatory) at the World Health Assembly in 2010 as an invaluable guide to global ethical recruitment for health professionals [1]. The Code aims to promote self-sustainability amongst developed countries rather than reliance on health professionals from developing countries. Despite some valid guidelines, the Code remains voluntary and difficult to monitor. The research is also consistent with Australia’s public policy commitment to promote the health of Australia’s population in terms of accessibility and equality to health whilst at the same time nurturing foreign relations with source countries.

“*In particular, the Secretaries and Ministers welcomed Australia’s commitment to assist the Philippines to upgrade qualifications of Philippine nurses to meet Australia’s market requirements*” (Joint Ministerial Statement, 9 October 2008 Manila).

We turn now to the empirical study of the source country experiences, perspectives, and recommendations.

## Methods

Data were collected through semi-structured focus group interviews with key informants in the Philippines. Assistance was provided by the Centre for Asian Integrity and Anticorruption Program, Institute for Ethics Governance and Law, Manila, who liaised with key institutions. After extensive communication, institutions were purposively selected based on the research teams’ prior knowledge and experience which included urban, rural, public, and private institutions. Key informants were identified in their area of expertise in education and health. Invitees were contacted by email and telephone to participate in interviews. Participants were purposively selected in order to garner varied perspectives on the consequences of nurse migration for the Philippines education and health sectors. Participants and/or institutions were not paid for their time. Responses from the focus group interviews focused explicitly on the perspective of the institutions involved in the education and employment of Filipino nurses, not individual perceptions, and are not the views of nurses themselves. Demographic data were collected via the questionnaire and consent form, provided on the day of interviews.

Interviews were conducted by the Australian (n = 2) and Filipino (n = 1) researchers in December 2011 in a private boardroom in Makati City, Metropolitan Manila. If the nominated invitee was unable to attend, they were asked to send another informant (four potential interviewees from two organisations were unable to attend on the day due to unforeseen circumstances).

We conducted focus group interviews with ten key informants from nine leading Filipino organisations (Table 1), which were allocated into one of two groups: i) education providers, including public and private universities and colleges, and ii) health providers, including both government (Department of Health) and the private sector.

**Table 1:** **Key informant groups and institutions**

	Key informant group	Organisation	Role of organisation
1	Health	St Luke’s Medical Center	Private hospital in the Philippines which provides state-of-the-art healthcare and fuses the expertise of locally- and internationally-trained doctors and medical professionals
2	Health	University of the Philippines & Philippines Department of Health	Public sector: provides quality health care, regulates all health services and products, and is the over-all technical authority on health
3	Health	Department of Science & Technology, Government of the Philippines	Public sector: providing central direction, leadership, and coordination of all scientific and technological activities, and of formulating policies, programs, and projects to support national development
4	Health	Human Resources for Health Network, Philippines Department of Health	Public sector: unit of the Department of Health which aims to provide equitable access for all people to an adequately trained, skilled, and supported health workforce
5	Education	University of Asia and Pacific	Private, not-for-profit institution of higher learning that offers some of the most outstanding academic programs in Asia
6	Education	De La Salle College	Private Nursing School offering Bachelor of Science in Nursing and Bachelor of Science in Midwifery
7	Education	St Paul University, Manila	Private Nursing School offering Bachelor of Science in Nursing and Bachelor of Science in Midwifery
8	Education	University of the Philippines	Government School of Nursing a University of the Philippines department which offers nursing courses and degrees to students.
9	Education	University of Santo Tomas	Catholic University in the Philippines

Organisations which participated in the education group (n = 5) included the University of Asia and the Pacific, De La Salle College, St Paul University (Manila), University of Philippines, and University of Santo Tomas. The health focus group (n = 4) included St Luke’s Medical Sector, the Department of Science and Technology, Human Resources for Health Network, and the Philippines Department of Health.

Formal ethical approval for the project was obtained from the Gold Coast Human Research Ethics Committee, Queensland, Australia. All participants were informed about the study, participant questions were answered, and consent obtained. All interviews were conducted in English and were digitally recorded and transcribed by a bi-lingual transcriber. The interview schedules were *a priori* coded, which are categories or codes that are developed before examining the data [22]. *A priori* coding was important for the interview schedule so that both focus groups were reliably presented the same structure and questions. *A priori* codes were discussed with colleagues on the research team who agreed on the following codes; health workforce migration, Filipino nursing education and standards, healthcare standards, and communication and ethical recruitment with particular emphasis on Australia. Opportunities to provide comments and feedback were also given. Qualitative content and thematic analysis was undertaken. Transcripts were read several times manually using highlighter and post-it notes for greater understanding of the text, key words, phrases, and patterns. A second stage of analysis was then undertaken using an Excel workbook with a coding framework which searched the transcripts, line by line, for recurrent patterns and alike or divergent quotes, opinions, and perspectives. For each informant, quotes were coded and sorted [23] into themes to identify differences and similarities between the Education and Health focus groups, and when the categories were exhausted, the codes were condensed into three broader themes of perceptions, experiences, and recommendations. One emergent issue, volunteerism, was noted. Intracoder reliability was achieved through the use of a single coder and peers on the project providing further direction to analyse key areas [9].

## Results

The principal themes identified were health workforce migration perceptions, quality and standards, and recommendations regarding health workforce migration. We turn first to Education.

### Education focus group

#### Health workforce migration perceptions

Informants from the group indicated that the United States was the preferred destination for Filipino nurse migration rather than Australia. Reasons the group highlighted included the ease of transition as Filipino nurses that pass the National Council Licensure Examination are eligible and registered to work in the United States without the need for clinical experience or additional nursing coursework. Migration to Australia was viewed as expensive for a Filipino nurse as nurses are required to undertake bridging courses and pass difficult English examinations. Family migration was considered an important issue by all in-formants.

“*Essentially, if I were a graduate nurse, I would have three options. Number one, I would see the US wherein, I don’t even need clinical experience........The next option is Middle East.......you don’t have to pay taxes....however you don’t bring your families…*” (University of Asia and the Pacific).

“*When migrants going to Australia will be allowed to bring families, it is a big help, it is a big advantage*” (University of Santo Tomas).

One organisation felt the Philippines was disadvantaged if a Filipino graduated from a state- or nationally-funded University as the government finances the cost of that education. If the nurse leaves for abroad then the beneficiaries are foreigners who did not invest in that education.

Difficulties were noted in the management and data collection regarding private migration. One informant suggested improved international management of global and international nurse recruitment. A country-to-country or government-to-government agreement was suggested by two key informants with the Labour Attaché’ of Australia nominated to assist in cooperation and implementation. Three informants then suggested that the Department of Labor and Employment, the Philippines Overseas Employment Administration, or the Overseas Workers Welfare Association should monitor labour flows. Two informants felt that nurses did not feel the presence of the Philippines Department of Health, so their involvement should be minimal. However, an informant suggested the Department of Health could have a larger role in managing the push factors that lead Filipino nurses to migrate.

#### Quality and standards

Negative consequences of outward migration from the Philippines were identified by the group, where experienced lecturers and professors at tertiary institutions migrated shortly after obtaining adequate experience. In the health sector, this high turnover rate burdens junior staff and hospitals, so top colleges and hospitals now implement a two-year contract to prevent Filipinos from leaving for abroad without serving their country first. Head nurses are the first to leave as they have clinical experience, which makes it easier for them to migrate, resulting in a high turnover rate of senior nursing staff (University of Santo Tomas, University of the Philippines, De Salle College).

“*And there could be some impact in the institution………the nurse is the head nurse of a certain hospital and then goes to another country. So what happens in the hospital where she* [was] *working? Suddenly you don’t have a head nurse. Sometimes they go without even notifying.*” (University of the Philippines).

The final Nursing Licensure Exam in the Philippines assists with maintaining quality and standards in nursing education and poor performing schools are evident from these results, which the group collectively agreed upon. Two informants supported the idea of establishing nursing education equivalency to an international standard. One informant raised concerns surrounding the cost of Australian nursing bridging courses which can deter registered nurses from the Philippines from working in the profession. Inability to register as a nurse also encourages Filipino nurses to work in a different profession or lower paid assistant jobs when they arrive in a foreign country and can result in skills wastage.

#### Recommendations regarding health workforce migration

Pronunciation was a concern amongst the group, especially Australian slang.

“*Because your “today” is* [sounds like] *“to die”…*” (St Paul University).

One informant suggested implementing orientation programs in the Philippines to assist nurses with the language-cultural transition to Australia, while another informant requested clear requirements for migration to Australia. One informant suggested government agencies might assist with the cost of bridging programs and referred to the Japanese Philippines Economic Partnership Agreement (JPEPA) as an example. Informants stated the JPEPA is a trade agreement between the Philippines and Japan. Most informants agreed that the JPEPA was not an attractive model of agreement for Filipino nurses, as learning the Japanese language was too difficult and near impossible to pass the exam.

“*Because with the experience with Japan, there were 200 nurses who tried to run there but only 1 passed the exam…*” (St Paul University).

All participants posited that a sustainable system of agreement was needed between both countries by Australian institutions offering faculty training to the education sector, such as educating nursing academics to the PhD level, since the majority only complete a Master degree. All informants in the focus group welcomed this suggestion. Key informants also acknowledged the risks associated with junior or inexperienced Filipino graduate nurses providing care for Filipino patients, as senior staff migrate abroad.

“*We* [nurses] *were like three months on the ward, and then we were fast to go to the emergency room too – so all the mistakes happen.*” (Educator at the University of Asia and the Pacific).

“*And the patients, they* [nurse] *endanger the lives.* [All yes in unison]” (University of Asia and the Pacific, De La Salle College, St Paul University, University of the Philippines, University of Santo Tomas).

Mutual benefits were suggested in terms of financial aid, scholarships, or faculty exchanges to encourage brain circulation, where an experienced Filipino nurse working abroad can return to the Philippines with new skills and assist with teaching/workshops through short term contracts. Two informants disagreed with return migration as hospitals were not willing to employ the older, experienced nurse for their skills as it was too costly for the hospital. It was considered by informants that the Philippines economy could not compete financially to retain nurses in the home country.

“*Well, there’s a real disparity in terms of pay.*” (University of the Philippines).

“*The irony is, we actually have a lot* [of nurses] *in the Philippines......there are no jobs.*” (University of Asia and the Pacific).

### Health focus group

#### Health workforce migration perceptions

One informant emphasised that the negative social consequences for family life, cultural, national, and ethical dimensions surrounding Filipino nurse migration were only now coming to light. One informant also acknowledged that females were predominantly migrating as caregivers.

“*The social impact, maybe the earlier years we don’t notice that. But now we can see what has happened to marriages, to parent-children relationships.*” (Human Resources for Health, Department of Health, University of the Philippines).

“*Overseas contractual workers......to be dominantly female. And the disaggregation of that would find them as caregivers…*” (Human Resources for Health, Department of Health, University of the Philippines).

Informants recognised the importance of monitoring systems for global workers and the welfare of Filipinos and applauded the establishment of the Commission on Filipinos Overseas. One informant did not support permanent migration but agreed that family migration was a great advantage for nurses to take their children abroad, but also reduces the likelihood of return migration. Participants were eager to develop an ethical system of migration to Australia as they viewed out-migration from the Philippines as inevitable. Beneficial aspects of migration from an economic perspective, including remittances were acknowledged. Most informants viewed the opportunity for Filipinos to migrate in a positive light.

“*So, we anticipate if there is a good ethical* [laughs] *thing of this migration to Australia, we don’t want to look at it as a disadvantage…*” (St Luke’s Medical Centre).

Nursing education improved, according to one key informant, whereby additional units of study were incorporated into the curricula such as transcultural aspects for those who wish to go abroad. Consequently, Filipino nurses want to exit the country once they have gained experience, which necessitates training new registered nurses every two years in the hospital setting, adding costs for training. To reduce this problem, a two-year contract is now in place for major hospitals which allows sufficient time for hospitals to recoup training costs. The cost of nursing education is borne by families in the private sector, while State Universities have incorporated a voluntary self-service where nurses serve the home country before working elsewhere. The nurses must sign a two-year contract and if they wish to leave abroad before the two-years, then the cost of training is to be returned back to the institution.

#### Quality and standards

All informants agreed that nursing education standards varied with the mushrooming of new schools of nursing to meet the demand for nurse training. However, the consensus was that top schools had maintained their standards. The Commission on Higher Education is gradually closing poor performing schools, which was agreed to be a positive step amongst the group.

Every informant in the health group agreed that Filipino nurses migrate abroad for socio-economic and financial reasons and that the Philippines is unlikely to be internationally competitive in the foreseeable future.

“*You can think of the basic wage of a nurse in the Philippines, it will definitely be not competitive to what they will earn abroad or in other countries.*” (Department of Science and Technology, Government of the Philippines).

An oversupply of nurses in the Philippines in conjunction with the global financial crisis and subsequent recession has led to a phenomenon referred to as ‘volunteerism.’ Informants stated that volunteers were able to get certificates for service in the hope of eventually applying for paid work elsewhere in the Philippines or abroad.

“*So what happens is that they* [volunteers] *become part of the staffing, which is not supposed to be, but they don’t get paid. After three or six months, they get* [volunteer] *certificates.*” (St Luke’s Medical Centre).

Nurses also choose to work in other fields until an opening for a nurse is available.

“*And there’s a problem, where do you place them* [nurses]*? .....you hear of reports on them going to areas unrelated to nursing like BPO* [Business Process Outsourcing] *and call centres* [in the Philippines]”. (Department of Science and Technology, Government of the Philippines).

Inadequate funding and other healthcare resources and issues were also mentioned.

“*We don’t say that the extra nurses here, the surplus nurses here are not needed in the country. In fact, they are needed but we do not have enough resources to put them in place in the health sector.*” (Human Resources for Health Network, Department of Health).

#### Recommendations regarding health workforce migration

The group unanimously supported the development of bilateral agreements.

“*So I think the issue of reciprocal benefits, reciprocal income, mutual benefits, comes into the surface, philosophically, ethically and in terms of its pragmatic and operational dimensions. It’s in the foreground now for us to attend to this developed-developing country relationship.*” (University of the Philippines & Department of Health).

Communication skills were not seen as a problem as Filipino nurses are taught using American-based textbooks. An important factor to consider during ethical agreements or models is to ensure integration of Filipino nurses into the recipient country and possible methods to encourage return migration to the Philippines on short-term contracts.

## Discussion

This research brings to light the perceptions and experiences of Filipinos on the ground following the Global Financial Crisis. Apparent from all key informants was that the migration of nurses is unlikely to abate and retention is challenging as a consequence of global financial, economical, and social pressures. Thus, the Philippines Government will continue to support emigration. Freedom of movement is an individual right [2], and with greater earning potential in developed countries there will be a constant pull of the best-qualified nurses out of the Philippines. Differences in salary are enormous, where a registered nurse will start at an income of $6,000 per month in Australia [24], in contrast to $100 to $180 per month in the Philippines [25]. Minimising this wage gap is impractical and migration enables Filipino nurses to send remittances back home, assisting families, and contributing to the economy. Remittances are a large source of income for the Philippines and other developing countries. The World Bank estimated that the Philippines received $23 billion in remittances in 2010 [26]. Only 3.8% of Philippines GDP was allocated to health expenditure to more recently 4.6% in 2012 [27].

This study highlights concerns surrounding volunteerism, a trend that has escalated in the last 5 years in the wake of the global financial crisis in the Philippines*.* The global financial crisis slowed international recruitment of nurses, which also exaggerated the oversupply of nurses in the Philippines. Volunteerism transpired where the Philippines experienced an oversupply of graduate nurses, without employment opportunities. Education focus group informants reported that graduate and registered nurses paid a fee to hospitals in the Philippines for nurses to work as volunteers, when they essentially became part of the workforce but without pay. The Department of Health was quoted reporting “*we’re also looking at how volunteerism can be part of a solution—how volunteers can be compensated, if at all*” [28]; this implies the persistence of a volunteer system in the Philippines. Jumilla [29] reports that some hospitals claim ‘training programs’ instead of stating volunteerism-for-a-fee. Despite the need for additional nurses, there is a lack of resources from the Philippines Government to support nurse positions. Numerous websites also promote volunteer positions for health professionals in rural areas of the Philippines, reinforcing the lack of resources for community clinics. Situations of underemployment are evident in many developing countries. In South Africa, 35,000 registered nurses are unemployed despite 32,000 nurse vacancies [30]. An oversupply of inexperienced nurses and volunteerism in the Philippines are negative consequences of out-migration.

The findings from the research also support the literature that highly qualified and experienced nurses are the first to leave for abroad. This has a negative effect on health care standards as hospitals tend to fill gaps with inexperienced and junior staff. Cheng [28] reports that 20 nursing schools failed to produce a single student who could pass the final Nursing Board examination in the Philippines. This further impedes the quality of care provided to the public, as senior nurses are not available to provide supervision and leadership. These common concerns were raised in both focus group interviews, where the departure of experienced nurses from the education and hospital sectors puts additional pressure on already overworked staff. This also encourages Filipino nurses to work abroad where the workload is less [30] and earning potential and opportunities for career development are much greater. Rural and remote areas in the Philippines are severely affected by this chain of movement, where a lack of experienced health workers has led to hospital closures and the maldistribution of skills and services [14]. Lorenzo [6] reported that some hospitals have one nurse to 60 patient ratios in the Philippines, hindering public access to quality health care and thus reducing the likelihood of achieving the Millennium Development Goals.

All informants concurred that there are varying standards of teaching between institutions (including hospitals) throughout the Philippines. An increased international demand for nurses has led to a mushrooming of nursing schools, and a reduction in the quality of nursing training and standards [31]. By 2005, 450 nursing schools offered Bachelor of Science in Nursing courses [15], but the annual number of nurses passing the Nursing Board examination declined to 54% between 2001 and 2004 [32]. The overall pass mark in the licensure examination indicates that the quality of nursing education has declined [31]. This can also be attributed to the limited training positions in hospitals for clinical placements combined with a lack of experienced senior nurses in the country to educate the next generation. Brush and Sochalski [31] also state that portable deans were evident in many schools whereby deans would take responsibilities over several schools with inadequate curricula, which is in accord with our results. Nursing education curricula are also shifting with inclusion of transcultural aspects for Filipinos wishing to work abroad, authenticating government and education systems adapting policies for the migration of health workers. Noting differences between top and poor training institutions, a registered migrant nurse is required to pass examinations and possibly complete bridging courses in the destination country. Informants also stated that repeating subjects wasted time and impeded earning potential while some nurses opted for lower paid positions. Working in lower paid positions further diminishes the disposable income for migrant nurses in the destination country [16]. Wages also differed for registered nurses depending on the country of training. In the UK, migrant nurses from non-European countries are required to undertake an ‘adaptation period’ before registration is granted [4], while in Canada, Filipinos are employed as ‘practical nurses’, receiving lower pay prior to registration [6].

English language is the major barrier to recruitment for health workers migrating to developed countries. Although many Filipinos speak English, challenges remain with English language proficiency in terms of reading, writing, expressing themselves articulately, and using host country terminology. Health informants lacked knowledge about the English examination requirements and considered Filipinos to ‘pick up’ the Australian English language without much difficulty. A communication problem is compounded by problems with Filipino nurses understanding accents and terminology and, conversely, source country patients understanding Filipino accents and terminology. Interestingly, not one informant was aware of English language barriers for Filipino nurses migrating to Australia. Martin et al. [33] confirm that Filipinos are competent at English, which makes them attractive nurses on a global scale. Hawthorne [34], however, highlights that English language testing and pre-migration qualifications assessment is a significant barrier for nurses migrating from non-English speaking countries and this includes the Philippines. In 2010, only 13% of Filipino nurses passed the Occupational English Test in Australia [34]. They fared worst in the reading sub-category, clearly this requires particular attention.

Family migration (where the entire family migrates with the Filipino nurse to the new destination country) was considered vital for nurses wishing to leave the Philippines, denoting the strong family culture [35]. Other researchers suggest that immigrant enclaves promote health because they contain sets of relationships, institutions, and social resources and prevent social disadvantages [36]. Despite informants acknowledging the economic benefits of health workforce migration, the socio-cultural aspects are less measureable. Perceptions of working abroad have become work aspirations of young Filipinos from all over the country according to informants, with labour migration likely to persist due to strengthening social networks and increased migration pathways.

With respect to the broader political economy, the Philippines Government purposely produces nurses for export in order to attract remittances from abroad [28, 32, 37]. The Association of Southeast Asian Nations, and other free trade agreements, will further facilitate the movement of health professionals between countries. Numerous specific country-country agreements included the United Kingdom, US, and Bahrain. The Saskatchewan memorandum of understanding was viewed as one of the favourable agreements by numerous participants. The Saskatchewan memorandum of understanding was an agreement between the Canadian Province of Saskatchewan and the Department of Labour and Employment of the Government of the Philippines from 2006 which streng-thens human resource issues in a more ethical manner through recruitment, employment conditions, training of workers, and the protection of Filipino nurses. In addition, Blank [37] reveals that the memorandum of understanding for Bahrain and the Philippines was consistent with ethical agreements with the incorporation of a scholarship program and academic mentoring to develop the Philippines health sector. Surprisingly, the global movement patterns of Filipino nurses were not discussed by any of the informant groups where a Filipino nurse is likely to work and migrate to several countries [9]. Many Filipino nurses shift to the Middle East and then permanently move to the UK or to the US or Australia, where they are likely to settle. Funds for employing additional nurses have not increased by the Philippines Government, despite the booming population since the 90s and the increased need for nurses in hospitals. The Health Human Resource Development Master Plan (2005–2030) of the Philippines Department of Health aims to manage future health workforce issues through integrating organisational policies and improving distribution of health resources, including budgeting and health outcomes for the Philippines health sector.

It is important to consider the limitations of this research. One was time constraints, which may have restricted participants during the discussion. Focus group interviews were the preferred method of data collection for this section of our study as the team was able to collect in depth opinions and information about the institutions they represent in a relatively short period of time. Education and health groups were more inclined to discuss issues they were concerned with, such as the tertiary sector or nurse ratios. It was not possible to include all data from each focus group and unfortunately two important organisations in the Philippines (Commission on Higher Education and Philippines Professional Regulation Commission) were unable to participate on the day. These organisations may have provided further insights which may be overlooked in our results.

## Conclusions

The economic benefits of health workforce migration on families and the economy are well documented [15, 17, 32]. An emerging body of research is recently focusing on the socio-cultural impact [16] on families and the broader society. Our study adds to the literature on health workforce migration perceptions and experiences and sheds new light on the concept of volunteerism that transpired as an influx of inexperienced nurses were left without job opportunities in the source country in the wake of the global financial crisis.

The study highlights key issues from leading Filipino organisations about the consequences of nurse migration, and how to ameliorate them. Health professional migration reflects the socio-political and economic situation in the Philippines in the context of global trends. Unable to match international salary standards, limited opportunities for employment and work overload in the Philippines pushes nurses to work abroad. Policy recommendations favoured by informants are threefold.

First, in the field of education, the study leads to the recommendation focusing on developing bilateral agreements [5] between source and recipient countries that include return in the form of either faculty exchanges at Universities, provision of supervision, and mentoring teachers to a PhD level.

Secondly, in the health sector, this study supports the recommendation providing orientation, bridging courses, and/or seminars in the Philippines. These recommendations have implications for planning, support, and orientation programmes for Filipino nurses. However, despite the positive recommendations for mutual benefits to the source country, the financial-legal-political challenges are far greater, for instance how would these measures be funded – by government, hospitals, or public or private institutions – and which organizations in the source country would be chosen for these exchanges or programs. The feasibility of such expected outcomes and proposed measures adds another level of complexity to migration.

Thirdly, with regard to the negative consequences associated with family dislocation, further research and dialogue is clearly needed concerning the social and cultural impacts of migration. Added quantitative and qualitative research is required to examine the experience, integration, and outcomes for Filipino nurses in developed countries.

Overall, this study highlights the dual discourse around migration, where, on the one hand, dire and negative consequences for education and health sectors occur with the policy of producing nurses for export in the Philippines. The education sector loses its capable educators and role models and the health sector has an oversupply of inexperienced nurses and families are dislocated by temporary migration of the female breadwinner, wife, and mother. On the other hand, nurses have the right to migrate abroad for better pay and work conditions, which adds to the complexity surrounding migration. This study also brought to light the increased reliance on so-called ‘volunteerism’ within the Philippines health sector. It is to be hoped that the policy recommendations gleaned from this empirical study will go some way towards ameliorating these negative consequences in the future.
